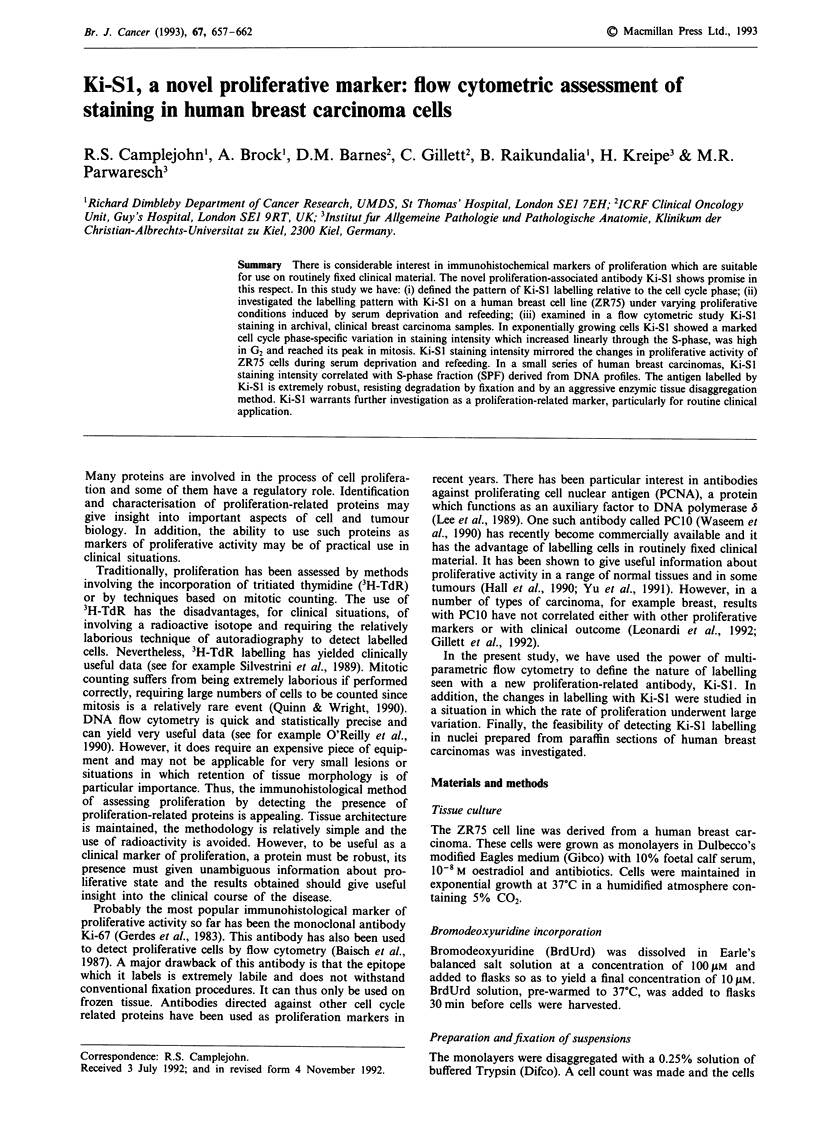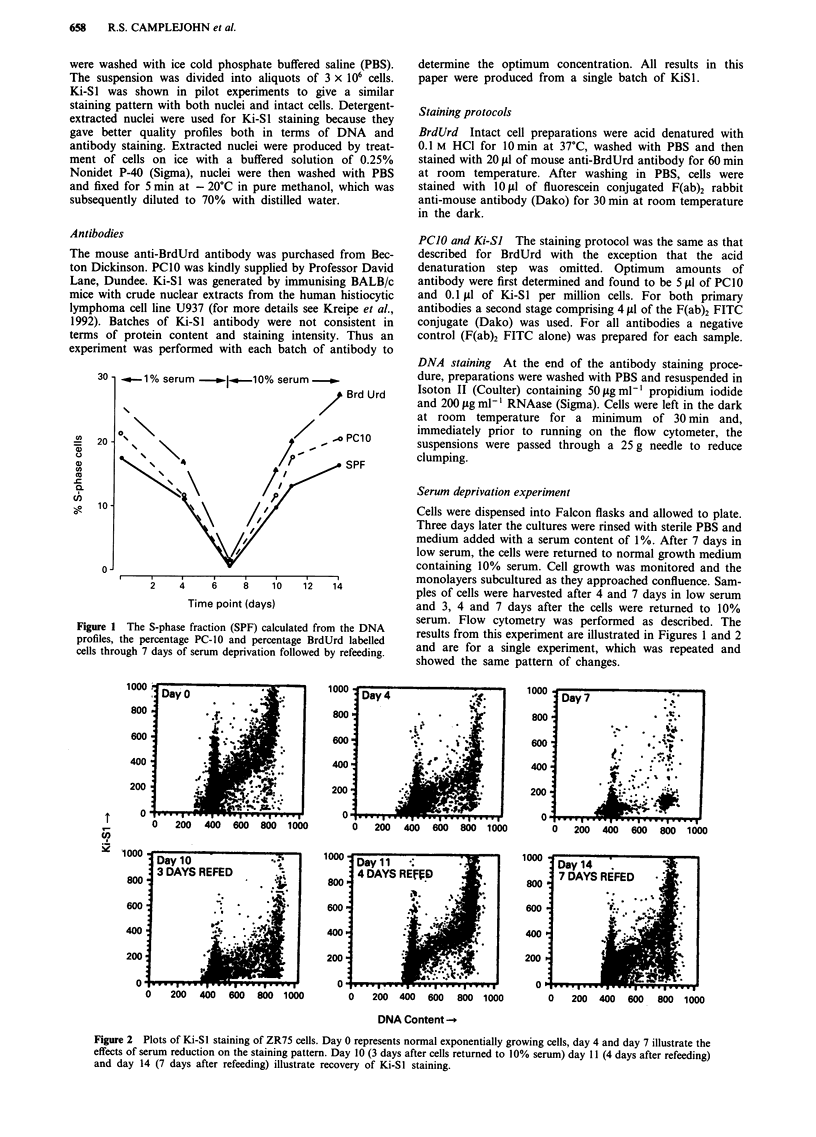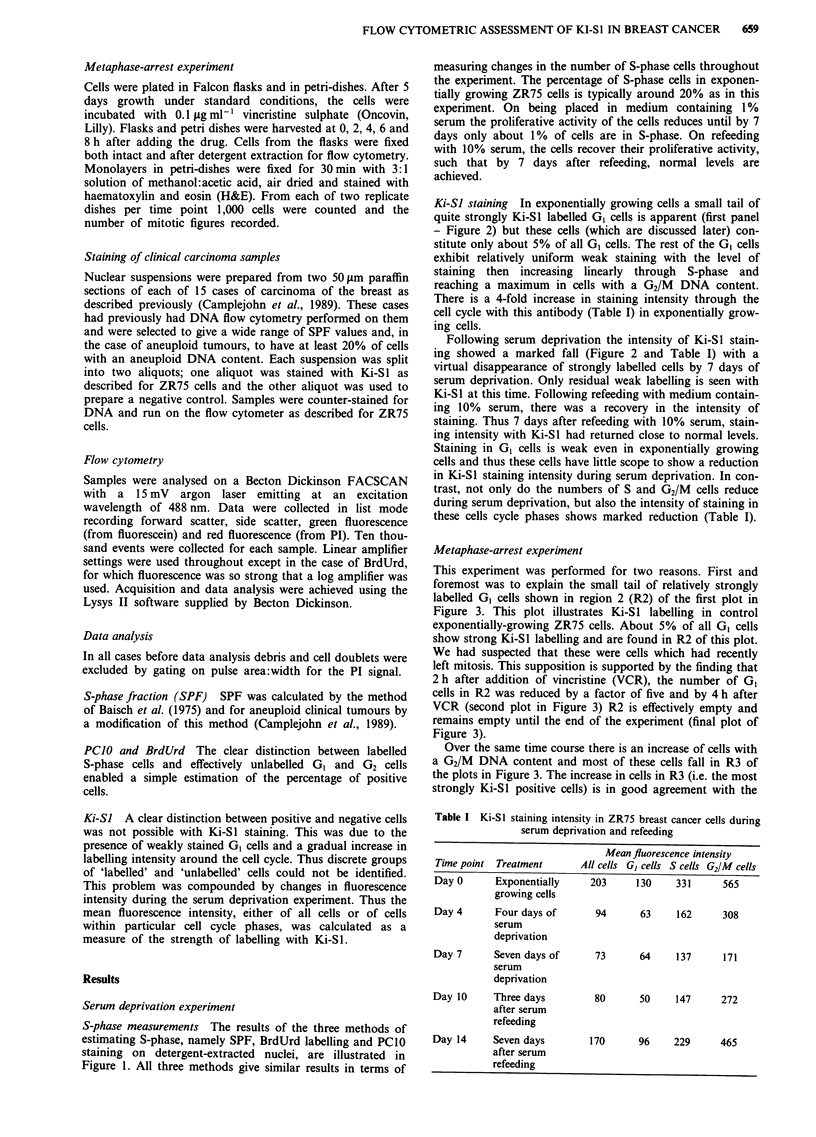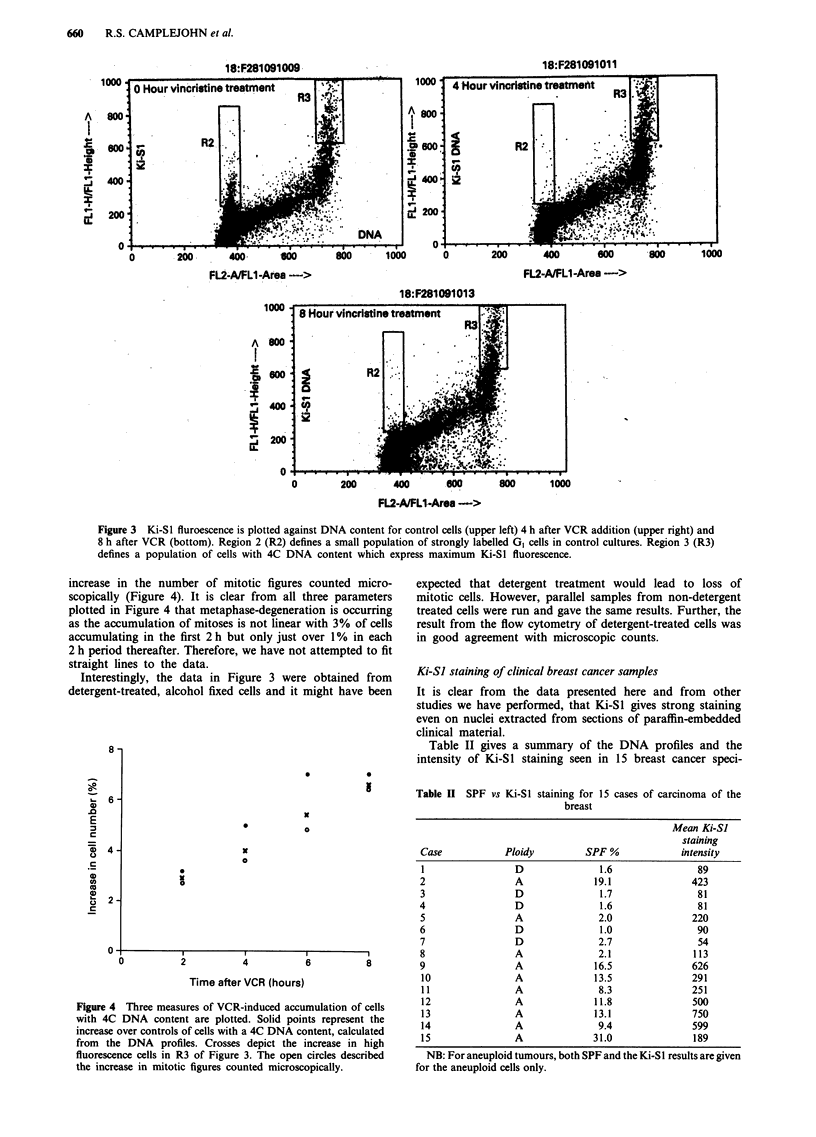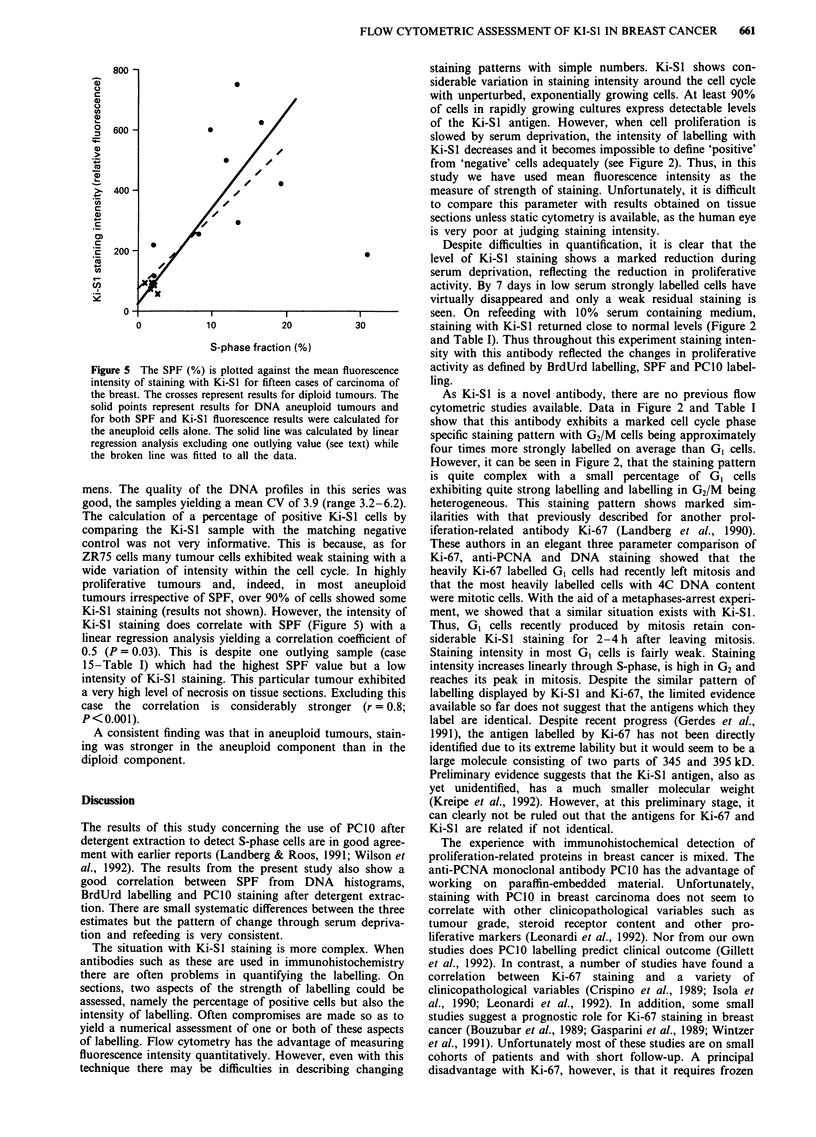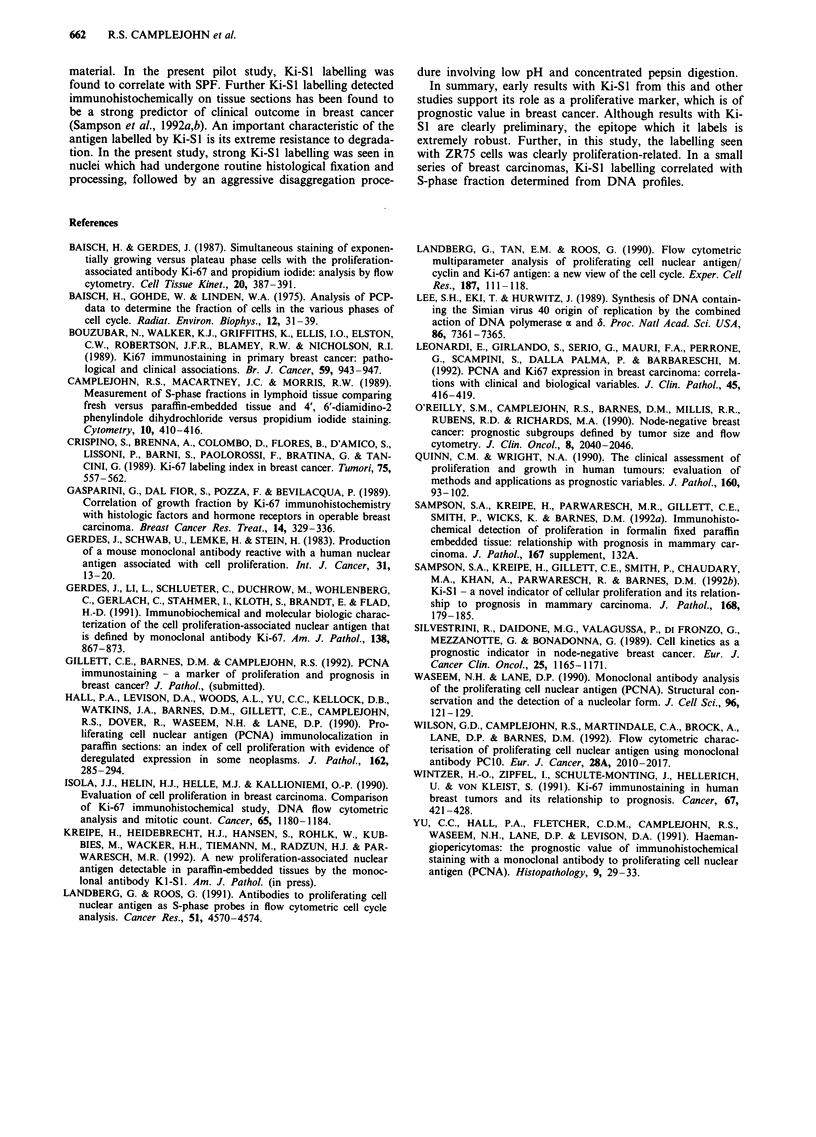# Ki-S1, a novel proliferative marker: flow cytometric assessment of staining in human breast carcinoma cells.

**DOI:** 10.1038/bjc.1993.122

**Published:** 1993-04

**Authors:** R. S. Camplejohn, A. Brock, D. M. Barnes, C. Gillett, B. Raikundalia, H. Kreipe, M. R. Parwaresch

**Affiliations:** Richard Dimbleby Department of Cancer Research, UMDS, St Thomas' Hospital, London.

## Abstract

There is considerable interest in immunohistochemical markers of proliferation which are suitable for use on routinely fixed clinical material. The novel proliferation-associated antibody Ki-S1 shows promise in this respect. In this study we have: (i) defined the pattern of Ki-S1 labelling relative to the cell cycle phase; (ii) investigated the labelling pattern with Ki-S1 on a human breast cell line (ZR75) under varying proliferative conditions induced by serum deprivation and refeeding; (iii) examined in a flow cytometric study Ki-S1 staining in archival, clinical breast carcinoma samples. In exponentially growing cells Ki-S1 showed a marked cell cycle phase-specific variation in staining intensity which increased linearly through the S-phase, was high in G2 and reached its peak in mitosis. Ki-S1 staining intensity mirrored the changes in proliferative activity of ZR75 cells during serum deprivation and refeeding. In a small series of human breast carcinoma, Ki-S1 staining intensity correlated with S-phase fraction (SPF) derived from DNA profiles. The antigen labelled by Ki-S1 is extremely robust, resisting degradation by fixation and by an aggressive enzymic tissue disaggregation method. Ki-S1 warrants further investigation as a proliferation-related marker, particularly for routine clinical application.


Br. J. Cancer (1993), 67, 657-662 © Macmillan Press Ltd., 1993

### Ki-S1, a novel proliferative marker: flow cytometric assessment of staining in human breast carcinoma cells

R.S. Camplejohn[1], A. Brock[1], D.M. Barnes[2], C. Gillett[2], B. Raikundalia[1], H. Kreipe[3] & M.R. Parwaresch[3]

[1]*Richard Dimbleby Department of Cancer Research, UMDS, St Thomas' Hospital, London SE1 7EH;* [2]*ICRF Clinical Oncology Unit, Guy's Hospital, London SE1 9RT, UK;* [3]*Institut fur Allgemeine Pathologie und Pathologische Anatomie, Klinikum der Christian-Albrechts-Universitat zu Kiel, 2300 Kiel, Germany.*

**Summary** There is considerable interest in immunohistochemical markers of proliferation which are suitable for use on routinely fixed clinical material. The novel proliferation-associated antibody Ki-S1 shows promise in this respect. In this study we have: (i) defined the pattern of Ki-S1 labelling relative to the cell cycle phase; (ii) investigated the labelling pattern with Ki-S1 on a human breast cell line (ZR75) under varying proliferative conditions induced by serum deprivation and refeeding; (iii) examined in a flow cytometric study Ki-S1 staining in archival, clinical breast carcinoma samples. In exponentially growing cells Ki-S1 showed a marked cell cycle phase-specific variation in staining intensity which increased linearly through the S-phase, was high in $G_2$ and reached its peak in mitosis. Ki-S1 staining intensity mirrored the changes in proliferative activity of ZR75 cells during serum deprivation and refeeding. In a small series of human breast carcinomas, Ki-S1 staining intensity correlated with S-phase fraction (SPF) derived from DNA profiles. The antigen labelled by Ki-S1 is extremely robust, resisting degradation by fixation and by an aggressive enzymic tissue disaggregation method. Ki-S1 warrants further investigation as a proliferation-related marker, particularly for routine clinical application.

Many proteins are involved in the process of cell proliferation and some of them have a regulatory role. Identification and characterisation of proliferation-related proteins may give insight into important aspects of cell and tumour biology. In addition, the ability to use such proteins as markers of proliferative activity may be of practical use in clinical situations.

Traditionally, proliferation has been assessed by methods involving the incorporation of tritiated thymidine ($^3$H-TdR) or by techniques based on mitotic counting. The use of $^3$H-TdR has the disadvantages, for clinical situations, of involving a radioactive isotope and requiring the relatively laborious technique of autoradiography to detect labelled cells. Nevertheless, $^3$H-TdR labelling has yielded clinically useful data (see for example Silvestrini *et al.*, 1989). Mitotic counting suffers from being extremely laborious if performed correctly, requiring large numbers of cells to be counted since mitosis is a relatively rare event (Quinn & Wright, 1990). DNA flow cytometry is quick and statistically precise and can yield very useful data (see for example O'Reilly *et al.*, 1990). However, it does require an expensive piece of equipment and may not be applicable for very small lesions or situations in which retention of tissue morphology is of particular importance. Thus, the immunohistological method of assessing proliferation by detecting the presence of proliferation-related proteins is appealing. Tissue architecture is maintained, the methodology is relatively simple and the use of radioactivity is avoided. However, to be useful as a clinical marker of proliferation, a protein must be robust, its presence must given unambiguous information about proliferative state and the results obtained should give useful insight into the clinical course of the disease.

Probably the most popular immunohistological marker of proliferative activity so far has been the monoclonal antibody Ki-67 (Gerdes *et al.*, 1983). This antibody has also been used to detect proliferative cells by flow cytometry (Baisch *et al.*, 1987). A major drawback of this antibody is that the epitope which it labels is extremely labile and does not withstand conventional fixation procedures. It can thus only be used on frozen tissue. Antibodies directed against other cell cycle related proteins have been used as proliferation markers in recent years. There has been particular interest in antibodies against proliferating cell nuclear antigen (PCNA), a protein which functions as an auxiliary factor to DNA polymerase $\delta$ (Lee *et al.*, 1989). One such antibody called PC10 (Waseem *et al.*, 1990) has recently become commercially available and it has the advantage of labelling cells in routinely fixed clinical material. It has been shown to give useful information about proliferative activity in a range of normal tissues and in some tumours (Hall *et al.*, 1990; Yu *et al.*, 1991). However, in a number of types of carcinoma, for example breast, results with PC10 have not correlated either with other proliferative markers or with clinical outcome (Leonardi *et al.*, 1992; Gillett *et al.*, 1992).

In the present study, we have used the power of multiparametric flow cytometry to define the nature of labelling seen with a new proliferation-related antibody, Ki-S1. In addition, the changes in labelling with Ki-S1 were studied in a situation in which the rate of proliferation underwent large variation. Finally, the feasibility of detecting Ki-S1 labelling in nuclei prepared from paraffin sections of human breast carcinomas was investigated.

### Materials and methods

### *Tissue culture*

The ZR75 cell line was derived from a human breast carcinoma. These cells were grown as monolayers in Dulbecco's modified Eagles medium (Gibco) with 10% foetal calf serum, $10^{-8}$ M oestradiol and antibiotics. Cells were maintained in exponential growth at 37°C in a humidified atmosphere containing 5% $CO_2$.

### *Bromodeoxyuridine incorporation*

Bromodeoxyuridine (BrdUrd) was dissolved in Earle's balanced salt solution at a concentration of 100 $\mu$M and added to flasks so as to yield a final concentration of 10 $\mu$M. BrdUrd solution, pre-warmed to 37°C, was added to flasks 30 min before cells were harvested.

### *Preparation and fixation of suspensions*

The monolayers were disaggregated with a 0.25% solution of buffered Trypsin (Difco). A cell count was made and the cells

Correspondence: R.S. Camplejohn.
Received 3 July 1992; and in revised form 4 November 1992.

were washed with ice cold phosphate buffered saline (PBS). The suspension was divided into aliquots of $3 \times 10^6$ cells. Ki-S1 was shown in pilot experiments to give a similar staining pattern with both nuclei and intact cells. Detergent-extracted nuclei were used for Ki-S1 staining because they gave better quality profiles both in terms of DNA and antibody staining. Extracted nuclei were produced by treatment of cells on ice with a buffered solution of 0.25% Nonidet P-40 (Sigma), nuclei were then washed with PBS and fixed for 5 min at $-20^{\circ}C$ in pure methanol, which was subsequently diluted to 70% with distilled water.

### *Antibodies*

The mouse anti-BrdUrd antibody was purchased from Becton Dickinson. PC10 was kindly supplied by Professor David Lane, Dundee. Ki-S1 was generated by immunising BALB/c mice with crude nuclear extracts from the human histiocytic lymphoma cell line U937 (for more details see Kreipe *et al.*, 1992). Batches of Ki-S1 antibody were not consistent in terms of protein content and staining intensity. Thus an experiment was performed with each batch of antibody to determine the optimum concentration. All results in this paper were produced from a single batch of KiS1.

**Figure 1** The S-phase fraction (SPF) calculated from the DNA profiles, the percentage PC-10 and percentage BrdUrd labelled cells through 7 days of serum deprivation followed by refeeding.

### *Staining protocols*

*BrdUrd* Intact cell preparations were acid denatured with 0.1 M HCl for 10 min at 37°C, washed with PBS and then stained with 20 μl of mouse anti-BrdUrd antibody for 60 min at room temperature. After washing in PBS, cells were stained with 10 μl of fluorescein conjugated $F(ab)_2$ rabbit anti-mouse antibody (Dako) for 30 min at room temperature in the dark.

*PC10 and Ki-S1* The staining protocol was the same as that described for BrdUrd with the exception that the acid denaturation step was omitted. Optimum amounts of antibody were first determined and found to be 5 μl of PC10 and 0.1 μl of Ki-S1 per million cells. For both primary antibodies a second stage comprising 4 μl of the $F(ab)_2$ FITC conjugate (Dako) was used. For all antibodies a negative control ($F(ab)_2$ FITC alone) was prepared for each sample.

*DNA staining* At the end of the antibody staining procedure, preparations were washed with PBS and resuspended in Isoton II (Coulter) containing 50 $\mu g\ ml^{-1}$ propidium iodide and 200 $\mu g\ ml^{-1}$ RNAase (Sigma). Cells were left in the dark at room temperature for a minimum of 30 min and, immediately prior to running on the flow cytometer, the suspensions were passed through a 25 g needle to reduce clumping.

### *Serum deprivation experiment*

Cells were dispensed into Falcon flasks and allowed to plate. Three days later the cultures were rinsed with sterile PBS and medium added with a serum content of 1%. After 7 days in low serum, the cells were returned to normal growth medium containing 10% serum. Cell growth was monitored and the monolayers subcultured as they approached confluence. Samples of cells were harvested after 4 and 7 days in low serum and 3, 4 and 7 days after the cells were returned to 10% serum. Flow cytometry was performed as described. The results from this experiment are illustrated in Figures 1 and 2 and are for a single experiment, which was repeated and showed the same pattern of changes.

**Figure 2** Plots of Ki-S1 staining of ZR75 cells. Day 0 represents normal exponentially growing cells, day 4 and day 7 illustrate the effects of serum reduction on the staining pattern. Day 10 (3 days after cells returned to 10% serum) day 11 (4 days after refeeding) and day 14 (7 days after refeeding) illustrate recovery of Ki-S1 staining.

*Metaphase-arrest experiment*

Cells were plated in Falcon flasks and in petri-dishes. After 5 days growth under standard conditions, the cells were incubated with 0.1 $\mu g\,ml^{-1}$ vincristine sulphate (Oncovin, Lilly). Flasks and petri dishes were harvested at 0, 2, 4, 6 and 8 h after adding the drug. Cells from the flasks were fixed both intact and after detergent extraction for flow cytometry. Monolayers in petri-dishes were fixed for 30 min with 3:1 solution of methanol:acetic acid, air dried and stained with haematoxylin and eosin (H&E). From each of two replicate dishes per time point 1,000 cells were counted and the number of mitotic figures recorded.

*Staining of clinical carcinoma samples*

Nuclear suspensions were prepared from two 50 $\mu$m paraffin sections of each of 15 cases of carcinoma of the breast as described previously (Camplejohn *et al.*, 1989). These cases had previously had DNA flow cytometry performed on them and were selected to give a wide range of SPF values and, in the case of aneuploid tumours, to have at least 20% of cells with an aneuploid DNA content. Each suspension was split into two aliquots; one aliquot was stained with Ki-S1 as described for ZR75 cells and the other aliquot was used to prepare a negative control. Samples were counter-stained for DNA and run on the flow cytometer as described for ZR75 cells.

*Flow cytometry*

Samples were analysed on a Becton Dickinson FACSCAN with a 15 mV argon laser emitting at an excitation wavelength of 488 nm. Data were collected in list mode recording forward scatter, side scatter, green fluorescence (from fluorescein) and red fluorescence (from PI). Ten thousand events were collected for each sample. Linear amplifier settings were used throughout except in the case of BrdUrd, for which fluorescence was so strong that a log amplifier was used. Acquisition and data analysis were achieved using the Lysys II software supplied by Becton Dickinson.

*Data analysis*

In all cases before data analysis debris and cell doublets were excluded by gating on pulse area:width for the PI signal.

*S-phase fraction (SPF)* SPF was calculated by the method of Baisch *et al.* (1975) and for aneuploid clinical tumours by a modification of this method (Camplejohn *et al.*, 1989).

*PC10 and BrdUrd* The clear distinction between labelled S-phase cells and effectively unlabelled $G_1$ and $G_2$ cells enabled a simple estimation of the percentage of positive cells.

*Ki-S1* A clear distinction between positive and negative cells was not possible with Ki-S1 staining. This was due to the presence of weakly stained $G_1$ cells and a gradual increase in labelling intensity around the cell cycle. Thus discrete groups of 'labelled' and 'unlabelled' cells could not be identified. This problem was compounded by changes in fluorescence intensity during the serum deprivation experiment. Thus the mean fluorescence intensity, either of all cells or of cells within particular cell cycle phases, was calculated as a measure of the strength of labelling with Ki-S1.

### Results

*Serum deprivation experiment*

*S-phase measurements* The results of the three methods of estimating S-phase, namely SPF, BrdUrd labelling and PC10 staining on detergent-extracted nuclei, are illustrated in Figure 1. All three methods give similar results in terms of measuring changes in the number of S-phase cells throughout the experiment. The percentage of S-phase cells in exponentially growing ZR75 cells is typically around 20% as in this experiment. On being placed in medium containing 1% serum the proliferative activity of the cells reduces until by 7 days only about 1% of cells are in S-phase. On refeeding with 10% serum, the cells recover their proliferative activity, such that by 7 days after refeeding, normal levels are achieved.

*Ki-S1 staining* In exponentially growing cells a small tail of quite strongly Ki-S1 labelled $G_1$ cells is apparent (first panel – Figure 2) but these cells (which are discussed later) constitute only about 5% of all $G_1$ cells. The rest of the $G_1$ cells exhibit relatively uniform weak staining with the level of staining then increasing linearly through S-phase and reaching a maximum in cells with a $G_2/M$ DNA content. There is a 4-fold increase in staining intensity through the cell cycle with this antibody (Table I) in exponentially growing cells.

Following serum deprivation the intensity of Ki-S1 staining showed a marked fall (Figure 2 and Table I) with a virtual disappearance of strongly labelled cells by 7 days of serum deprivation. Only residual weak labelling is seen with Ki-S1 at this time. Following refeeding with medium containing 10% serum, there was a recovery in the intensity of staining. Thus 7 days after refeeding with 10% serum, staining intensity with Ki-S1 had returned close to normal levels. Staining in $G_1$ cells is weak even in exponentially growing cells and thus these cells have little scope to show a reduction in Ki-S1 staining intensity during serum deprivation. In contrast, not only do the numbers of S and $G_2/M$ cells reduce during serum deprivation, but also the intensity of staining in these cells cycle phases shows marked reduction (Table I).

*Metaphase-arrest experiment*

This experiment was performed for two reasons. First and foremost was to explain the small tail of relatively strongly labelled $G_1$ cells shown in region 2 (R2) of the first plot in Figure 3. This plot illustrates Ki-S1 labelling in control exponentially-growing ZR75 cells. About 5% of all $G_1$ cells show strong Ki-S1 labelling and are found in R2 of this plot. We had suspected that these were cells which had recently left mitosis. This supposition is supported by the finding that 2 h after addition of vincristine (VCR), the number of $G_1$ cells in R2 was reduced by a factor of five and by 4 h after VCR (second plot in Figure 3) R2 is effectively empty and remains empty until the end of the experiment (final plot of Figure 3).

Over the same time course there is an increase of cells with a $G_2/M$ DNA content and most of these cells fall in R3 of the plots in Figure 3. The increase in cells in R3 (i.e. the most strongly Ki-S1 positive cells) is in good agreement with the

**Table I** Ki-S1 staining intensity in ZR75 breast cancer cells during serum deprivation and refeeding

| *Time point* | *Treatment* | *Mean fluorescence intensity* *All cells* | *$G_1$ cells* | *S cells* | *$G_2/M$ cells* |
|---|---|---|---|---|---|
| Day 0 | Exponentially growing cells | 203 | 130 | 331 | 565 |
| Day 4 | Four days of serum deprivation | 94 | 63 | 162 | 308 |
| Day 7 | Seven days of serum deprivation | 73 | 64 | 137 | 171 |
| Day 10 | Three days after serum refeeding | 80 | 50 | 147 | 272 |
| Day 14 | Seven days after serum refeeding | 170 | 96 | 229 | 465 |

**Figure 3** Ki-S1 fluroescence is plotted against DNA content for control cells (upper left) 4 h after VCR addition (upper right) and 8 h after VCR (bottom). Region 2 (R2) defines a small population of strongly labelled $G_1$ cells in control cultures. Region 3 (R3) defines a population of cells with 4C DNA content which express maximum Ki-S1 fluorescence.

increase in the number of mitotic figures counted microscopically (Figure 4). It is clear from all three parameters plotted in Figure 4 that metaphase-degeneration is occurring as the accumulation of mitoses is not linear with 3% of cells accumulating in the first 2 h but only just over 1% in each 2 h period thereafter. Therefore, we have not attempted to fit straight lines to the data.

Interestingly, the data in Figure 3 were obtained from detergent-treated, alcohol fixed cells and it might have been expected that detergent treatment would lead to loss of mitotic cells. However, parallel samples from non-detergent treated cells were run and gave the same results. Further, the result from the flow cytometry of detergent-treated cells was in good agreement with microscopic counts.

**Figure 4** Three measures of VCR-induced accumulation of cells with 4C DNA content are plotted. Solid points represent the increase over controls of cells with a 4C DNA content, calculated from the DNA profiles. Crosses depict the increase in high fluorescence cells in R3 of Figure 3. The open circles described the increase in mitotic figures counted microscopically.

*Ki-S1 staining of clinical breast cancer samples*

It is clear from the data presented here and from other studies we have performed, that Ki-S1 gives strong staining even on nuclei extracted from sections of paraffin-embedded clinical material.

Table II gives a summary of the DNA profiles and the intensity of Ki-S1 staining seen in 15 breast cancer speci-

**Table II** SPF *vs* Ki-S1 staining for 15 cases of carcinoma of the breast

| *Case* | *Ploidy* | *SPF %* | *Mean Ki-S1 staining intensity* |
|---|---|---|---|
| 1 | D | 1.6 | 89 |
| 2 | A | 19.1 | 423 |
| 3 | D | 1.7 | 81 |
| 4 | D | 1.6 | 81 |
| 5 | A | 2.0 | 220 |
| 6 | D | 1.0 | 90 |
| 7 | D | 2.7 | 54 |
| 8 | A | 2.1 | 113 |
| 9 | A | 16.5 | 626 |
| 10 | A | 13.5 | 291 |
| 11 | A | 8.3 | 251 |
| 12 | A | 11.8 | 500 |
| 13 | A | 13.1 | 750 |
| 14 | A | 9.4 | 599 |
| 15 | A | 31.0 | 189 |

NB: For aneuploid tumours, both SPF and the Ki-S1 results are given for the aneuploid cells only.

**Figure 5** The SPF (%) is plotted against the mean fluorescence intensity of staining with Ki-S1 for fifteen cases of carcinoma of the breast. The crosses represent results for diploid tumours. The solid points represent results for DNA aneuploid tumours and for both SPF and Ki-S1 fluorescence results were calculated for the aneuploid cells alone. The solid line was calculated by linear regression analysis excluding one outlying value (see text) while the broken line was fitted to all the data.

mens. The quality of the DNA profiles in this series was good, the samples yielding a mean CV of 3.9 (range 3.2–6.2). The calculation of a percentage of positive Ki-S1 cells by comparing the Ki-S1 sample with the matching negative control was not very informative. This is because, as for ZR75 cells many tumour cells exhibited weak staining with a wide variation of intensity within the cell cycle. In highly proliferative tumours and, indeed, in most aneuploid tumours irrespective of SPF, over 90% of cells showed some Ki-S1 staining (results not shown). However, the intensity of Ki-S1 staining does correlate with SPF (Figure 5) with a linear regression analysis yielding a correlation coefficient of 0.5 ($P = 0.03$). This is despite one outlying sample (case 15–Table I) which had the highest SPF value but a low intensity of Ki-S1 staining. This particular tumour exhibited a very high level of necrosis on tissue sections. Excluding this case the correlation is considerably stronger ($r = 0.8$; $P < 0.001$).

A consistent finding was that in aneuploid tumours, staining was stronger in the aneuploid component than in the diploid component.

### Discussion

The results of this study concerning the use of PC10 after detergent extraction to detect S-phase cells are in good agreement with earlier reports (Landberg & Roos, 1991; Wilson *et al.*, 1992). The results from the present study also show a good correlation between SPF from DNA histograms, BrdUrd labelling and PC10 staining after detergent extraction. There are small systematic differences between the three estimates but the pattern of change through serum deprivation and refeeding is very consistent.

The situation with Ki-S1 staining is more complex. When antibodies such as these are used in immunohistochemistry there are often problems in quantifying the labelling. On sections, two aspects of the strength of labelling could be assessed, namely the percentage of positive cells but also the intensity of labelling. Often compromises are made so as to yield a numerical assessment of one or both of these aspects of labelling. Flow cytometry has the advantage of measuring fluorescence intensity quantitatively. However, even with this technique there may be difficulties in describing changing staining patterns with simple numbers. Ki-S1 shows considerable variation in staining intensity around the cell cycle with unperturbed, exponentially growing cells. At least 90% of cells in rapidly growing cultures express detectable levels of the Ki-S1 antigen. However, when cell proliferation is slowed by serum deprivation, the intensity of labelling with Ki-S1 decreases and it becomes impossible to define 'positive' from 'negative' cells adequately (see Figure 2). Thus, in this study we have used mean fluorescence intensity as the measure of strength of staining. Unfortunately, it is difficult to compare this parameter with results obtained on tissue sections unless static cytometry is available, as the human eye is very poor at judging staining intensity.

Despite difficulties in quantification, it is clear that the level of Ki-S1 staining shows a marked reduction during serum deprivation, reflecting the reduction in proliferative activity. By 7 days in low serum strongly labelled cells have virtually disappeared and only a weak residual staining is seen. On refeeding with 10% serum containing medium, staining with Ki-S1 returned close to normal levels (Figure 2 and Table I). Thus throughout this experiment staining intensity with this antibody reflected the changes in proliferative activity as defined by BrdUrd labelling, SPF and PC10 labelling.

As Ki-S1 is a novel antibody, there are no previous flow cytometric studies available. Data in Figure 2 and Table I show that this antibody exhibits a marked cell cycle phase specific staining pattern with $G_2$/M cells being approximately four times more strongly labelled on average than $G_1$ cells. However, it can be seen in Figure 2, that the staining pattern is quite complex with a small percentage of $G_1$ cells exhibiting quite strong labelling and labelling in $G_2$/M being heterogeneous. This staining pattern shows marked similarities with that previously described for another proliferation-related antibody Ki-67 (Landberg *et al.*, 1990). These authors in an elegant three parameter comparison of Ki-67, anti-PCNA and DNA staining showed that the heavily Ki-67 labelled $G_1$ cells had recently left mitosis and that the most heavily labelled cells with 4C DNA content were mitotic cells. With the aid of a metaphases-arrest experiment, we showed that a similar situation exists with Ki-S1. Thus, $G_1$ cells recently produced by mitosis retain considerable Ki-S1 staining for 2–4 h after leaving mitosis. Staining intensity in most $G_1$ cells is fairly weak. Staining intensity increases linearly through S-phase, is high in $G_2$ and reaches its peak in mitosis. Despite the similar pattern of labelling displayed by Ki-S1 and Ki-67, the limited evidence available so far does not suggest that the antigens which they label are identical. Despite recent progress (Gerdes *et al.*, 1991), the antigen labelled by Ki-67 has not been directly identified due to its extreme lability but it would seem to be a large molecule consisting of two parts of 345 and 395 kD. Preliminary evidence suggests that the Ki-S1 antigen, also as yet unidentified, has a much smaller molecular weight (Kreipe *et al.*, 1992). However, at this preliminary stage, it can clearly not be ruled out that the antigens for Ki-67 and Ki-S1 are related if not identical.

The experience with immunohistochemical detection of proliferation-related proteins in breast cancer is mixed. The anti-PCNA monoclonal antibody PC10 has the advantage of working on paraffin-embedded material. Unfortunately, staining with PC10 in breast carcinoma does not seem to correlate with other clinicopathological variables such as tumour grade, steroid receptor content and other proliferative markers (Leonardi *et al.*, 1992). Nor from our own studies does PC10 labelling predict clinical outcome (Gillett *et al.*, 1992). In contrast, a number of studies have found a correlation between Ki-67 staining and a variety of clinicopathological variables (Crispino *et al.*, 1989; Isola *et al.*, 1990; Leonardi *et al.*, 1992). In addition, some small studies suggest a prognostic role for Ki-67 staining in breast cancer (Bouzubar *et al.*, 1989; Gasparini *et al.*, 1989; Wintzer *et al.*, 1991). Unfortunately most of these studies are on small cohorts of patients and with short follow-up. A principal disadvantage with Ki-67, however, is that it requires frozen material. In the present pilot study, Ki-S1 labelling was found to correlate with SPF. Further Ki-S1 labelling detected immunohistochemically on tissue sections has been found to be a strong predictor of clinical outcome in breast cancer (Sampson *et al.*, 1992*a,b*). An important characteristic of the antigen labelled by Ki-S1 is its extreme resistance to degradation. In the present study, strong Ki-S1 labelling was seen in nuclei which had undergone routine histological fixation and processing, followed by an aggressive disaggregation procedure involving low pH and concentrated pepsin digestion.

In summary, early results with Ki-S1 from this and other studies support its role as a proliferative marker, which is of prognostic value in breast cancer. Although results with Ki-S1 are clearly preliminary, the epitope which it labels is extremely robust. Further, in this study, the labelling seen with ZR75 cells was clearly proliferation-related. In a small series of breast carcinomas, Ki-S1 labelling correlated with S-phase fraction determined from DNA profiles.

### References

BAISCH, H. & GERDES, J. (1987). Simultaneous staining of exponentially growing versus plateau phase cells with the proliferation-associated antibody Ki-67 and propidium iodide: analysis by flow cytometry. *Cell Tissue Kinet.*, **20**, 387-391.

BAISCH, H., GOHDE, W. & LINDEN, W.A. (1975). Analysis of PCP-data to determine the fraction of cells in the various phases of cell cycle. *Radiat. Environ. Biophys.*, **12**, 31-39.

BOUZUBAR, N., WALKER, K.J., GRIFFITHS, K., ELLIS, I.O., ELSTON, C.W., ROBERTSON, J.F.R., BLAMEY, R.W. & NICHOLSON, R.I. (1989). Ki67 immunostaining in primary breast cancer: pathological and clinical associations. *Br. J. Cancer*, **59**, 943-947.

CAMPLEJOHN, R.S., MACARTNEY, J.C. & MORRIS, R.W. (1989). Measurement of S-phase fractions in lymphoid tissue comparing fresh versus paraffin-embedded tissue and 4', 6'-diamidino-2 phenylindole dihydrochloride versus propidium iodide staining. *Cytometry*, **10**, 410-416.

CRISPINO, S., BRENNA, A., COLOMBO, D., FLORES, B., D'AMICO, S., LISSONI, P., BARNI, S., PAOLOROSSI, F., BRATINA, G. & TANCINI, G. (1989). Ki-67 labeling index in breast cancer. *Tumori*, **75**, 557-562.

GASPARINI, G., DAL FIOR, S., POZZA, F. & BEVILACQUA, P. (1989). Correlation of growth fraction by Ki-67 immunohistochemistry with histologic factors and hormone receptors in operable breast carcinoma. *Breast Cancer Res. Treat.*, **14**, 329-336.

GERDES, J., SCHWAB, U., LEMKE, H. & STEIN, H. (1983). Production of a mouse monoclonal antibody reactive with a human nuclear antigen associated with cell proliferation. *Int. J. Cancer*, **31**, 13-20.

GERDES, J., LI, L., SCHLUETER, C., DUCHROW, M., WOHLENBERG, C., GERLACH, C., STAHMER, I., KLOTH, S., BRANDT, E. & FLAD, H.-D. (1991). Immunobiochemical and molecular biologic characterization of the cell proliferation-associated nuclear antigen that is defined by monoclonal antibody Ki-67. *Am. J. Pathol.*, **138**, 867-873.

GILLETT, C.E., BARNES, D.M. & CAMPLEJOHN, R.S. (1992). PCNA immunostaining – a marker of proliferation and prognosis in breast cancer? *J. Pathol.*, (submitted).

HALL, P.A., LEVISON, D.A., WOODS, A.L., YU, C.C., KELLOCK, D.B., WATKINS, J.A., BARNES, D.M., GILLETT, C.E., CAMPLEJOHN, R.S., DOVER, R., WASEEM, N.H. & LANE, D.P. (1990). Proliferating cell nuclear antigen (PCNA) immunolocalization in paraffin sections: an index of cell proliferation with evidence of deregulated expression in some neoplasms. *J. Pathol.*, **162**, 285-294.

ISOLA, J.J., HELIN, H.J., HELLE, M.J. & KALLIONIEMI, O.-P. (1990). Evaluation of cell proliferation in breast carcinoma. Comparison of Ki-67 immunohistochemical study, DNA flow cytometric analysis and mitotic count. *Cancer*, **65**, 1180-1184.

KREIPE, H., HEIDEBRECHT, H.J., HANSEN, S., ROHLK, W., KUBBIES, M., WACKER, H.H., TIEMANN, M., RADZUN, H.J. & PARWARESCH, M.R. (1992). A new proliferation-associated nuclear antigen detectable in paraffin-embedded tissues by the monoclonal antibody K1-S1. *Am. J. Pathol.* (in press).

LANDBERG, G. & ROOS, G. (1991). Antibodies to proliferating cell nuclear antigen as S-phase probes in flow cytometric cell cycle analysis. *Cancer Res.*, **51**, 4570-4574.

LANDBERG, G., TAN, E.M. & ROOS, G. (1990). Flow cytometric multiparameter analysis of proliferating cell nuclear antigen/cyclin and Ki-67 antigen: a new view of the cell cycle. *Exper. Cell Res.*, **187**, 111-118.

LEE, S.H., EKI, T. & HURWITZ, J. (1989). Synthesis of DNA containing the Simian virus 40 origin of replication by the combined action of DNA polymerase α and δ. *Proc. Natl Acad. Sci. USA*, **86**, 7361-7365.

LEONARDI, E., GIRLANDO, S., SERIO, G., MAURI, F.A., PERRONE, G., SCAMPINI, S., DALLA PALMA, P. & BARBARESCHI, M. (1992). PCNA and Ki67 expression in breast carcinoma: correlations with clinical and biological variables. *J. Clin. Pathol.*, **45**, 416-419.

O'REILLY, S.M., CAMPLEJOHN, R.S., BARNES, D.M., MILLIS, R.R., RUBENS, R.D. & RICHARDS, M.A. (1990). Node-negative breast cancer: prognostic subgroups defined by tumor size and flow cytometry. *J. Clin. Oncol.*, **8**, 2040-2046.

QUINN, C.M. & WRIGHT, N.A. (1990). The clinical assessment of proliferation and growth in human tumours: evaluation of methods and applications as prognostic variables. *J. Pathol.*, **160**, 93-102.

SAMPSON, S.A., KREIPE, H., PARWARESCH, M.R., GILLETT, C.E., SMITH, P., WICKS, K. & BARNES, D.M. (1992*a*). Immunohistochemical detection of proliferation in formalin fixed paraffin embedded tissue: relationship with prognosis in mammary carcinoma. *J. Pathol.*, **167** supplement, 132A.

SAMPSON, S.A., KREIPE, H., GILLETT, C.E., SMITH, P., CHAUDARY, M.A., KHAN, A., PARWARESCH, R. & BARNES, D.M. (1992*b*). Ki-S1 – a novel indicator of cellular proliferation and its relationship to prognosis in mammary carcinoma. *J. Pathol.*, **168**, 179-185.

SILVESTRINI, R., DAIDONE, M.G., VALAGUSSA, P., DI FRONZO, G., MEZZANOTTE, G. & BONADONNA, G. (1989). Cell kinetics as a prognostic indicator in node-negative breast cancer. *Eur. J. Cancer Clin. Oncol.*, **25**, 1165-1171.

WASEEM, N.H. & LANE, D.P. (1990). Monoclonal antibody analysis of the proliferating cell nuclear antigen (PCNA). Structural conservation and the detection of a nucleolar form. *J. Cell Sci.*, **96**, 121-129.

WILSON, G.D., CAMPLEJOHN, R.S., MARTINDALE, C.A., BROCK, A., LANE, D.P. & BARNES, D.M. (1992). Flow cytometric characterisation of proliferating cell nuclear antigen using monoclonal antibody PC10. *Eur. J. Cancer*, **28A**, 2010-2017.

WINTZER, H.-O., ZIPFEL, I., SCHULTE-MONTING, J., HELLERICH, U. & VON KLEIST, S. (1991). Ki-67 immunostaining in human breast tumors and its relationship to prognosis. *Cancer*, **67**, 421-428.

YU, C.C., HALL, P.A., FLETCHER, C.D.M., CAMPLEJOHN, R.S., WASEEM, N.H., LANE, D.P. & LEVISON, D.A. (1991). Haemangiopericytomas: the prognostic value of immunohistochemical staining with a monoclonal antibody to proliferating cell nuclear antigen (PCNA). *Histopathology*, **9**, 29-33.